# Identification and validation of differentially expressed genes in allergic asthma pathogenesis using whole-transcriptome sequencing

**DOI:** 10.3389/fmed.2025.1545095

**Published:** 2025-04-16

**Authors:** Fang Cui, Rui Liu, Li Wang, Jing He, Yanxia Xu

**Affiliations:** ^1^Department of Laboratory Medicine, Yanan University Affiliated Hospital, Yan'an, Shaanxi, China; ^2^Department of Respiratory and Critical Care Medicine, Yanan University Affiliated Hospital, Yan'an, Shaanxi, China

**Keywords:** allergic asthma, whole-transcriptome sequencing, differentially expressed genes, functional enrichment analysis, asthma pathogenesis

## Abstract

**Objective:**

This study aims to systematically identify differential gene expression profiles in patients with allergic asthma through whole-transcriptome sequencing and validate the role of these genes in asthma pathogenesis, thereby uncovering potential molecular mechanisms.

**Methods:**

This study recruited a cohort of 80 individuals diagnosed with allergic asthma and 40 healthy controls. RNA was extracted from both peripheral blood and airway samples, and sequencing was performed using the Illumina NovaSeq 6000 platform. Potential differential genes were confirmed through three independent techniques to validate the findings: quantitative reverse transcription polymerase chain reaction (qRT-PCR), immunohistochemistry, and Western blot analysis. Differential gene expression was analyzed using DESeq2 software, providing a rigorous statistical framework for RNA-Seq data interpretation. Gene Ontology (GO) enrichment analysis and Kyoto Encyclopedia of Genes and Genomes (KEGG) pathway analysis were employed to elucidate the biological significance of the differentially expressed genes, offering insights into the molecular mechanisms underlying allergic asthma.

**Results:**

Differential expression analysis identified multiple genes with significant differences between the patient and control groups. Inflammatory-related genes such as *IL1B*, *CCL17*, and *MUC5AC* were significantly upregulated in the patient group (*p* < 0.05), while regulatory immune factors such as *FOXP3* and *IFNG* showed significantly higher expression in the control group (*p* < 0.05). Functional enrichment analysis indicated that the differential genes were mainly enriched in immune response, T-cell activation, and *MAPK* signaling pathways. Experimental validation demonstrated consistency between transcriptomic data and RNA and protein expression levels, further supporting the involvement of these genes in asthma.

**Conclusion:**

Differential gene expression profiles play a crucial role in the pathogenesis of asthma. This study provides important evidence for understanding the molecular mechanisms of asthma and developing novel targeted therapeutic strategies.

## 1 Introduction

Asthma is a highly heterogeneous disease characterized by airway inflammation and remodeling, affecting over 300 million people worldwide (1). Allergic asthma leads to substantial morbidity and represents a major public health burden, with its impact escalating as the severity of the disease increases. Despite the availability of various medications for clinical treatment, the challenges of asthma recurrence and refractoriness remain prevalent, primarily due to its complex pathophysiological mechanisms (2). The characteristic pathological changes in asthma include airway inflammation, airway hyperresponsiveness, and remodeling processes. These changes are typically driven by abnormal activation of immune cells and excessive release of pro-inflammatory factors (3, 4). Although some progress has been made in understanding the molecular mechanisms of asthma, particularly in regulating inflammatory mediators and immune imbalance, numerous gene expression characteristics remain unexplored.

Previous studies have revealed the roles of certain inflammatory and immune regulatory factors in asthma pathogenesis (5, 6). However, systematic identification and validation of differentially expressed genes remain insufficient. Specifically, it is not yet fully understood which specific genes exhibit expression changes across various tissues and levels to drive disease progression in asthma (7). This gap in knowledge limits our comprehensive understanding of asthma’s pathological mechanisms and hinders the development of novel targeted therapies. Therefore, further investigation of transcriptome-wide gene expression profiles in asthma patients is imperative to elucidate its underlying molecular mechanisms and provide a theoretical foundation for future clinical interventions.

In this study, we systematically identified differentially expressed specific genes in allergic asthma patients through whole-transcriptome sequencing and validated key genes using qRT-PCR, immunohistochemistry, and Western blotting to clarify their roles in asthma pathogenesis. The findings of this study not only offer a novel perspective on the molecular mechanisms of asthma but also provide critical targets for the development of future therapeutic strategies, demonstrating significant clinical relevance and innovation.

## 2 Materials and methods

### 2.1 Study design

This study employed a prospective cohort design comprising an allergic asthma patient group (80 subjects) and a healthy control group (40 subjects). The objective was to identify and validate the role of differential gene expression characteristics in the pathogenesis of allergic asthma through whole-transcriptome sequencing. Participants in the allergic asthma group and the healthy control group were matched by age, gender, and ethnicity to minimize confounding factors. To further control potential confounders, all sample collection was conducted at the same time of day (early morning in a fasting state). A comprehensive medical history was obtained, and strict exclusion criteria were applied to ensure that participants had no other chronic conditions that could potentially influence gene expression. The study was conducted over 2 years, from July 2022 to July 2024, encompassing sample collection, sequencing, data analysis, and experimental validation.

### 2.2 Study subjects and sample collection

#### 2.2.1 Study population

**Figure 1:**
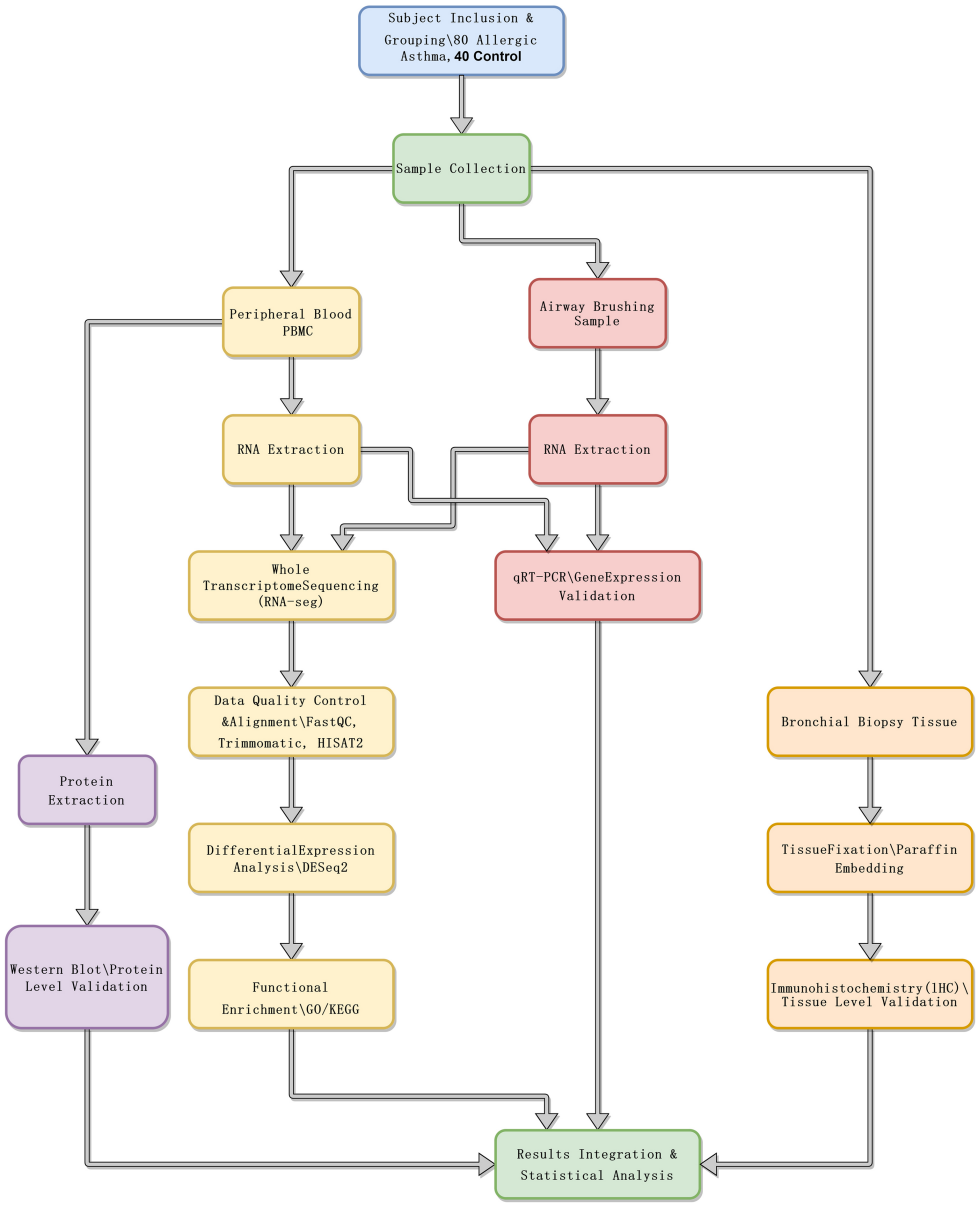
Experimental flow chart.

The study enrolled 80 patients with allergic asthma and 40 healthy controls. The diagnosis of allergic asthma was based on the Global Initiative for Asthma (GINA) Guidelines, 2021 Edition (8), and all participants were adults aged 18 to 60 years. Allergic asthma patients were confirmed positive for common allergens via skin prick tests or serum-specific IgE testing. Healthy controls were matched to the patient group in terms of age, gender, and ethnicity, and had no history of asthma or other respiratory diseases. Spirometry, total IgE levels, and blood eosinophil counts were also measured to characterize the participants further. Figure 1 presents a schematic diagram of the experimental workflow.

#### 2.2.2 Sample types and collection methods

##### 2.2.2.1 Peripheral blood sample collection

For all study participants, 10 mL of peripheral venous blood was collected in the early morning under fasting conditions using EDTA-coated anticoagulant tubes. Within 1 h of collection, peripheral blood mononuclear cells (PBMCs) were isolated using Ficoll density gradient centrifugation. The centrifugation conditions were set to 400 g for 30 min at room temperature, with no brake applied. Isolated PBMCs were washed twice with PBS immediately after separation and then stored at −80°C for subsequent RNA extraction.

##### 2.2.2.2 Airway brushing sample collection

A bronchoscopic examination was performed on a subset of participants, including 30 patients and 15 healthy controls, to collect airway epithelial cell samples. A disposable bronchial brush (Olympus BC-202D-2010) was used to gently brush the bronchial wall surface, with approximately 10 strokes at each site, to ensure adequate cell collection. Sampling sites included the main bronchi and lobar bronchi. The collected cells were immediately placed in 1 mL of RNAlater solution and stored at −80°C within 1 h of collection for later use. Sequencing was performed on both PBMCs and airway brushing samples.

### 2.3 RNA extraction and whole-transcriptome sequencing

#### 2.3.1 RNA extraction

##### 2.3.1.1 Peripheral blood mononuclear cells

After isolating peripheral blood mononuclear cells (PBMCs) using Ficoll density gradient centrifugation, total RNA was extracted using the reliable TRIzol reagent from Thermo Fisher Scientific. To assess the quality of our RNA, we measured its concentration and purity using the NanoDrop 2000 spectrophotometer, targeting an A260/A280 ratio between 1.8 and 2.1. To verify the integrity of our RNA, we performed gel electrophoresis using a 1.5% agarose gel, loading 1 μg of RNA per lane. The gel was run at 100 V for 30 min (9).

##### 2.3.1.2 Airway epithelial cells

The airway epithelial cells were preserved in RNAlater solution, and RNA extraction was subsequently performed using the RNeasy Mini Kit (Qiagen), adhering closely to the manufacturer’s protocols. The concentration and purity of the RNA were assessed using the NanoDrop 2000 spectrophotometer, and RNA integrity was further evaluated using the Bioanalyzer 2100 system (Agilent Technologies) to ensure sample quality (10).

#### 2.3.2 Whole-transcriptome sequencing

Whole-transcriptome sequencing was performed using the Illumina NovaSeq 6000 platform, with library preparation conducted using the NEBNext Ultra RNA Library Prep Kit (New England Biolabs) according to the manufacturer’s protocol. The target sequencing depth for each sample was set at 100 million paired-end reads (100 M PE) to ensure sufficient genomic coverage and sequencing accuracy (11). The quality control standard for sequencing data required a Q30 score of at least 85%, providing high-quality and reliable results. Library quality was assessed using the Qubit system and the Agilent Bioanalyzer 2100 to ensure compliance with sequencing requirements before initiating sequencing on the Illumina platform. Sequencing was performed for both PBMCs and airway brushing samples, and data were analyzed separately for each tissue type.

### 2.4 Bioinformatics analysis

#### 2.4.1 Data preprocessing

##### 2.4.1.1 Quality control

The raw sequencing data underwent initial quality control using FastQC, which assessed metrics such as base quality distribution and GC content.

##### 2.4.1.2 Data filtering

Low-quality reads and adapter sequences were removed using Trimmomatic software to retain high-quality reads for downstream analyses. The following specific parameters were applied:

LEADING: 20 and TRAILING: 20 to trim bases with quality scores below 20 from the ends of reads.
SLIDINGWINDOW: 4:20 to perform a sliding window quality check, trimming regions with average quality scores below 20 within a 4-base window.
MINLEN: 50 to discard reads shorter than 50 base pairs.

This filtering process ensured the removal of low-quality sequences, retaining only high-quality reads longer than 50 base pairs for accurate downstream analysis.”

#### 2.4.2 Differential expression analysis

##### 2.4.2.1 Alignment and quantification

The high-quality reads were aligned to the human reference genome (GRCh38) using HISAT2, with alignment parameters set to a maximum of 2 mismatches and a seed length of 20 bases to ensure accuracy. Following alignment, transcript assembly and quantification of gene expression levels were performed using StringTie, with parameters including a minimum transcript coverage threshold of 0.1 to ensure the reliability of expression levels. Default alignment settings were used to achieve maximum alignment rates and accuracy.

##### 2.4.2.2 Differential gene screening

Differential gene expression analysis was conducted using the DESeq2 package on normalized gene expression data. Genes were considered differentially expressed if they met the criteria of |log2 Fold Change| ≥ 1 and a False Discovery Rate (FDR) < 0.05, ensuring the statistical significance of the identified genes.

#### 2.4.3 Functional enrichment analysis

To explore the functional roles of the identified genes, we performed Gene Ontology (GO) and KEGG Pathway Enrichment Analysis. Differentially expressed genes (DEGs) were analyzed using the ClusterProfiler software, which facilitated the assessment of their enrichment across three distinct categories of GO: biological processes (BP), cellular components (CC), and molecular functions (MF) (12). We set a significance threshold at *p* < 0.05 to ensure our findings were robust and employed the Benjamini-Hochberg method to keep the false discovery rate (FDR) in check. Only pathways with an enrichment ratio exceeding 1.5 were considered significant for further analysis (13). For the KEGG pathway analysis, we referenced the human genome (GRCh38) and set a threshold of at least 10 genes per pathway to ensure the reliability of the results. This approach allowed us to uncover significant connections within the genetic landscape.

### 2.5 Experimental validation

#### 2.5.1 Quantitative real-time PCR

To validate our findings, we selected 10 candidate genes from the pool of differentially expressed genes for further analysis using quantitative real-time PCR (qRT-PCR). We converted 1 μg of total RNA into complementary DNA (cDNA) using the PrimeScript RT Reagent Kit (Takara). The subsequent step involved amplification using SYBR Green Master Mix (Applied Biosystems) on the ABI 7500 Real-Time PCR System. Each sample was analyzed in triplicate, with biological and technical replicates separated by 30-min intervals, ensuring consistent environmental conditions—such as temperature and reaction mix—throughout the process. GAPDH was used as the internal control gene, and relative gene expression levels were calculated using the ΔΔCt method.

#### 2.5.2 Immunohistochemistry and protein validation

##### 2.5.2.1 Immunohistochemistry

Using immunohistochemistry (IHC) to investigate protein expression, we examined bronchial biopsy tissues from 30 patients and 15 control subjects. The tissue samples were first fixed in formalin, processed into paraffin blocks, and sectioned into 4 μm slices. These sections were then deparaffinized and rehydrated, followed by antigen retrieval in a citrate buffer facilitated by a pressure cooker to enhance the detection of target proteins.

Once the nonspecific binding was minimized, the primary antibody (from Abcam, diluted 1:200) was added and incubated overnight at 4°C. The following day, the sections were incubated with HRP-conjugated secondary antibodies, followed by DAB staining for visualization and hematoxylin counterstaining for contrast. The blocking step lasted 1 h, while antigen retrieval was completed in 20 min. The primary antibody was incubated overnight at 4°C, and the secondary antibody was applied at room temperature for 1 h. The staining was then independently scored by two pathologists, who assessed the intensity and proportion of positively stained cells and averaged their scores to determine the final result.

##### 2.5.2.2 Western blotting

We focused on the total protein extracted from peripheral blood PBMCs using RIPA buffer in another research phase. Protein concentration was quantified using the BCA assay to ensure accurate amounts for our experiments. SDS-PAGE then separated equal protein quantities (30 μg per lane) on 12% polyacrylamide gels run at a constant voltage of 120 V for approximately 90 min.

The proteins were transferred to PVDF membranes using a constant current of 300 mA for 90 min. To block nonspecific binding, the membranes were incubated with 5% non-fat milk at room temperature for 1 h. Next, the primary antibody (from Abcam, diluted 1:1,000) was applied and incubated overnight at 4°C. The following day, HRP-conjugated secondary antibodies were introduced, and protein bands were detected using ECL and visualized with the ChemiDoc imaging system (Bio-Rad). *β*-actin was used as the internal control, and band intensities were analyzed using ImageJ software to complete the protein analysis.

### 2.6 Data analysis

The data were analyzed using SPSS version 26.0. Quantitative data are presented as mean ± standard deviation, and group comparisons were made using independent sample t-tests. Categorical data were analyzed with the chi-square test. qRT-PCR results were interpreted using the ΔΔCt method, and differences between multiple groups were assessed using one-way ANOVA. A *p*-value of less than 0.05 was deemed statistically significant.

## 3 Results

### 3.1 Sample characteristics and sequencing data quality

**Figure 2:**
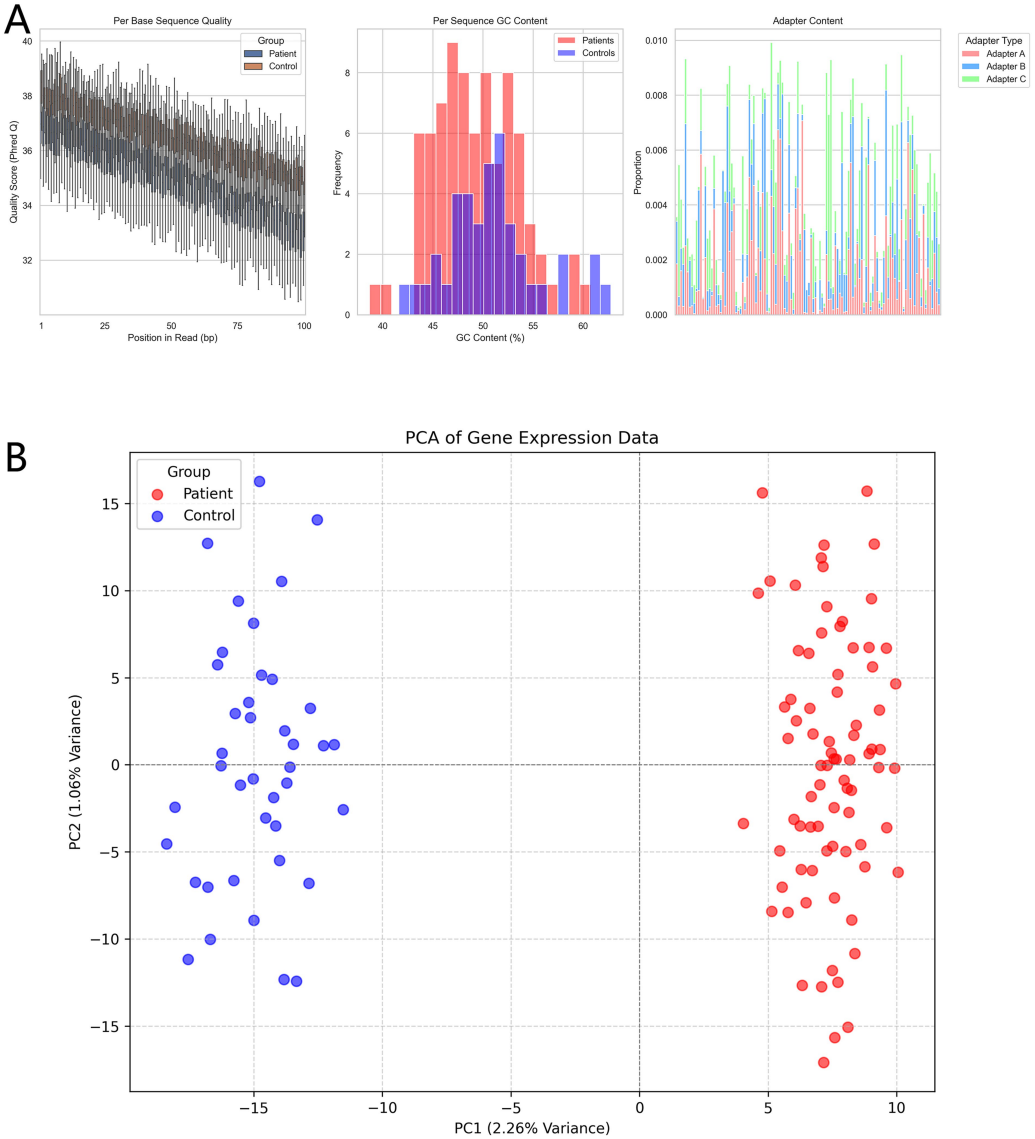
**(A)** Quality assessment of sequencing data (FastQC results). Boxplots show position-based Q-score variations for base quality; histograms compare the frequency distributions of GC content; stacked bar charts display the proportions of different types of adapter contamination. **(B)** Principal component analysis (PCA) shows distinct clustering of the patient and control groups based on gene expression profiles.

There were no statistically significant differences in baseline characteristics between the allergic asthma patient group and the healthy control group regarding age, gender, BMI, smoking history, or past medical history (*p* > 0.05), indicating comparability between the two groups. However, significant differences were observed in allergen types (*p* < 0.001), with a higher proportion of pollen and dust mite allergies in the patient group. Regarding treatment, 62.5% of asthma patients had received corticosteroid therapy (Table 1). Quality evaluation of the sequencing data showed similar base quality distributions between the patient and control groups, although the quality in the patient group was slightly lower (Figure 2A). GC content did not differ significantly between the two groups, and the overall distribution was as expected. Adapter contamination analysis revealed a low proportion of adapter sequences across all samples. Principal component analysis (PCA) revealed a clear separation between the patient and control groups based on gene expression profiles (Figure 2B). The first principal component (PC1) explained the largest variance, with the patient and control groups forming distinct clusters, reflecting significant intergroup differences and intragroup consistency.

**Table 1:** Baseline characteristics of the study population.

Baseline characteristics	Allergic asthma group (*n* = 80)	Healthy control group (*n* = 40)	*P*-value
Age (years, mean ± SD)	42.58 ± 11.22	40.35 ± 9.85	0.316
Gender (male, *n* (%))	38 (47.50%)	19 (47.50%)	1.000
BMI (kg/m^2^, mean ± SD)	25.76 ± 3.54	24.98 ± 3.17	0.285
Allergen type [*n* (%)]			
Pollen	45 (56.25%)	0 (0%)	<0.001
Dust mites	35 (43.75%)	0 (0%)	<0.001
Disease duration [years, median (IQR)]	8 (4–15)	N/A	-
Smoking history [*n* (%)]	28 (35.00%)	10 (25.00%)	0.278
Past medical history [*n* (%)]	24 (30.00%)	8 (20.00%)	0.262
Treatment [*n* (%)]			
Corticosteroid therapy	50 (62.50%)	N/A	-
No medication	30 (37.50%)	N/A	-
FEV₁% predicted (%, mean ± SD)	79.35 ± 6.42	89.22 ± 5.91	<0.001
FEV₁/FVC (%, mean ± SD)	70.14 ± 4.98	80.10 ± 3.44	<0.001
Total IgE [IU/mL, median (IQR)]	210.46 (158.81–282.33)	45.37 (30.52–59.69)	<0.001
Peripheral blood eosinophils (×10^9^/L, mean ± SD)	0.36 ± 0.04	0.20 ± 0.03	<0.001

### 3.2 Identification of differentially expressed genes

Differential expression analysis identified several genes with significantly altered expression between the patient and control groups. Upregulated genes, including those associated with inflammation and allergic responses, all had *p*-values < 0.05 and remained statistically significant after FDR correction (Table 2).

**Table 2:** List of differentially expressed genes.

Gene name	Log2 fold change	*P*-value	False discovery rate (FDR)
*IL1B*	1.84	0.003	0.011
*CCL17*	2.15	0.002	0.013
*POSTN*	1.52	0.008	0.019
*IL13*	1.97	0.004	0.015
*TNF*	1.38	0.012	0.022
*MUC5AC*	2.45	0.001	0.009
*ALOX5*	1.69	0.006	0.018
*SERPINB2*	1.28	0.014	0.026
*IFNG*	−1.52	0.009	0.021
*FOXP3*	−1.22	0.015	0.027
*STAT4*	−1.75	0.005	0.014
*IL10*	−1.41	0.011	0.023
*CXCL9*	−1.33	0.013	0.025
*GATA3*	−1.25	0.016	0.029
*ORMDL3*	−1.58	0.007	0.018

Differentially expressed genes were analyzed using DESeq2, with *P*-values and false discovery rates (FDRs) used to assess statistical significance.

**Figure 3:**
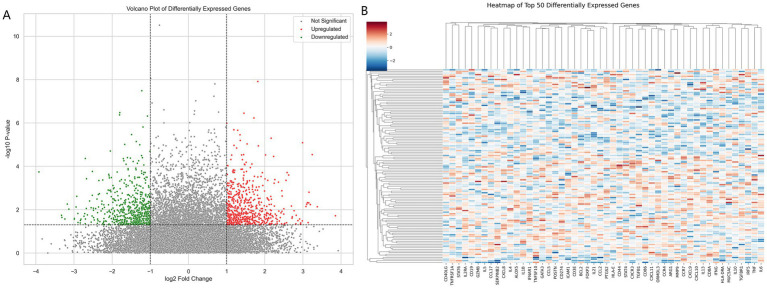
**(A)** Volcano plot of differentially expressed genes (DEGs) analyzed using DESeq2, based on log2 fold change and *p*-values, highlighting upregulated (red) and downregulated (green) genes. **(B)** Heatmap of the top 50 significantly differentially expressed genes, displaying standardized expression values (Z-scores) derived from DESeq2 analysis.

The analysis revealed significant gene expression changes between asthma patients and controls. Genes with significant upregulation and downregulation are highlighted in red and green in the volcano plot, respectively, while the majority of genes without significant differences are shown in gray (Figure 3A). Expression levels of differentially expressed genes showed clear clustering between the patient and control groups, with most patient samples exhibiting a high-expression pattern. Hierarchical clustering analysis further revealed distinct branches for patients and controls, indicating marked differences in gene expression profiles (Figure 3B).

### 3.3 Functional enrichment analysis of differentially expressed genes

GO functional enrichment analysis revealed that the differentially expressed genes were significantly enriched in biological processes (BP) such as immune response, inflammatory response, and T cell differentiation (Table 3). All enriched terms had *p*-values < 0.05, supporting the hypothesis that these genes are involved in asthma pathogenesis. These findings highlight the role of these processes in the development of asthma.

**Table 3:** Results of GO functional enrichment analysis.

GO term (name)	Category	Gene count	*P*-value	FDR-corrected value	Enrichment ratio
Immune response	BP	9	0.002	0.008	3.7
Inflammatory response	BP	11	0.001	0.006	4.1
Cell migration	BP	7	0.004	0.015	3.0
Cytokine activity	MF	6	0.008	0.018	2.8
Regulation of T cell differentiation	BP	8	0.003	0.011	3.3
Regulatory T cell differentiation	BP	6	0.005	0.016	3.2
Plasma membrane	CC	8	0.003	0.012	3.1
Protein binding	MF	10	0.007	0.02	2.9
Regulation of cell proliferation	BP	9	0.003	0.01	3.4
Extracellular matrix	CC	5	0.009	0.022	2.7
Signal transduction	BP	12	0.002	0.009	3.8
T cell activation	BP	10	0.004	0.014	3.5
Cell surface	CC	7	0.005	0.017	2.8
Enzyme activity regulation	MF	8	0.006	0.018	3.1
Regulation of cellular secretion	BP	9	0.003	0.011	3.3

GO enrichment analysis was conducted using the DAVID tool based on significantly differentially expressed genes. Significance was assessed using Fisher’s exact test, and results were corrected for multiple comparisons using the false discovery rate (FDR) method.

**Figure 4:**
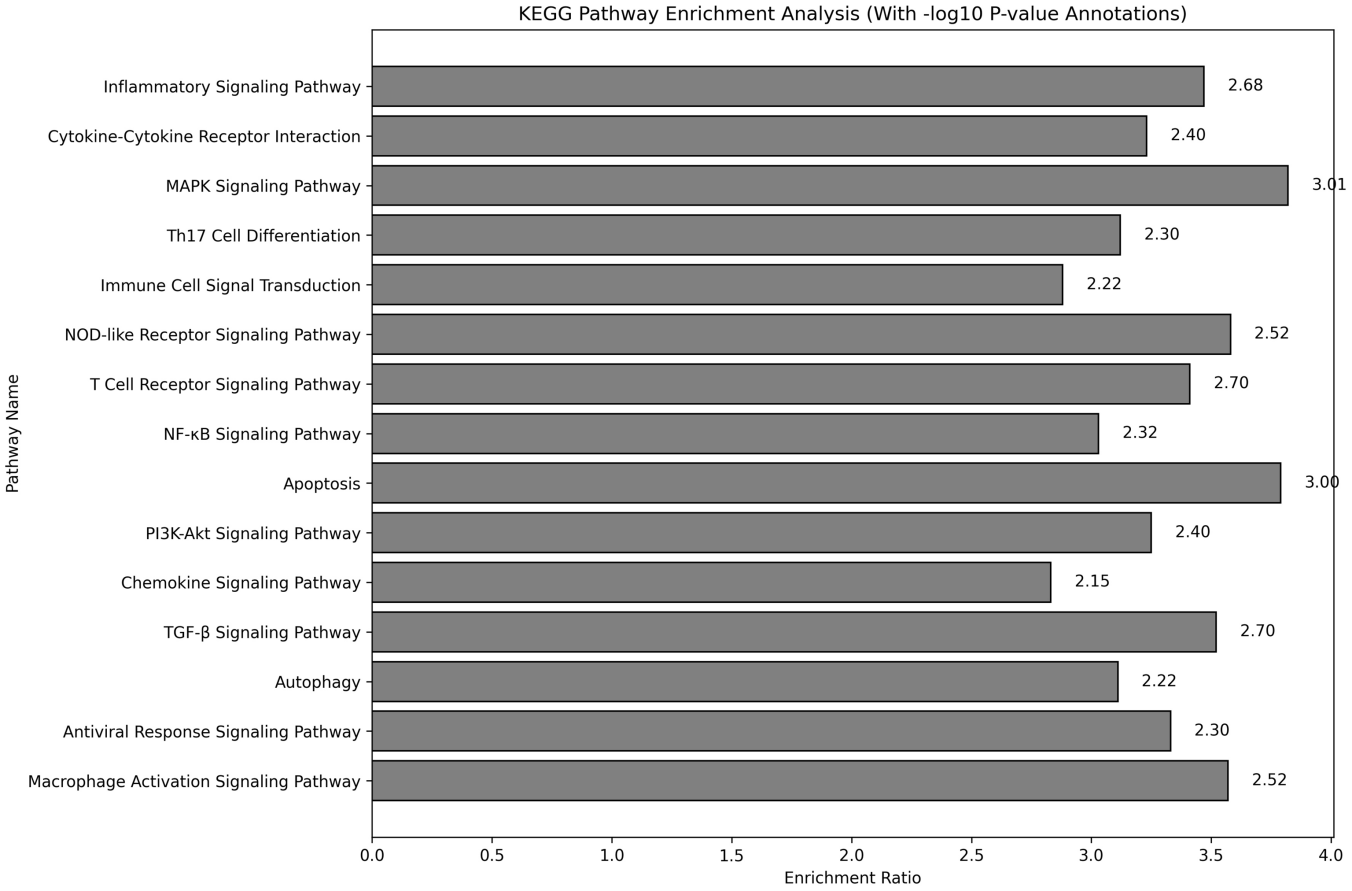
KEGG pathway enrichment analysis showing pathways significantly associated with differentially expressed genes. The analysis was conducted using the DAVID tool, with Fisher’s exact test assessing significance. −log10 *p*-values represent the significance level for each pathway.

KEGG pathway enrichment analysis indicated that the differentially expressed genes were significantly enriched in key pathways, including the inflammatory signaling pathway, MAPK signaling pathway, and PI3K-Akt signaling pathway (Figure 4). The enrichment ratios and −log10 *p*-values were statistically significant, suggesting that these pathways may play critical roles in asthma pathophysiology.

### 3.4 Validation of differentially expressed genes

**Figure 5:**
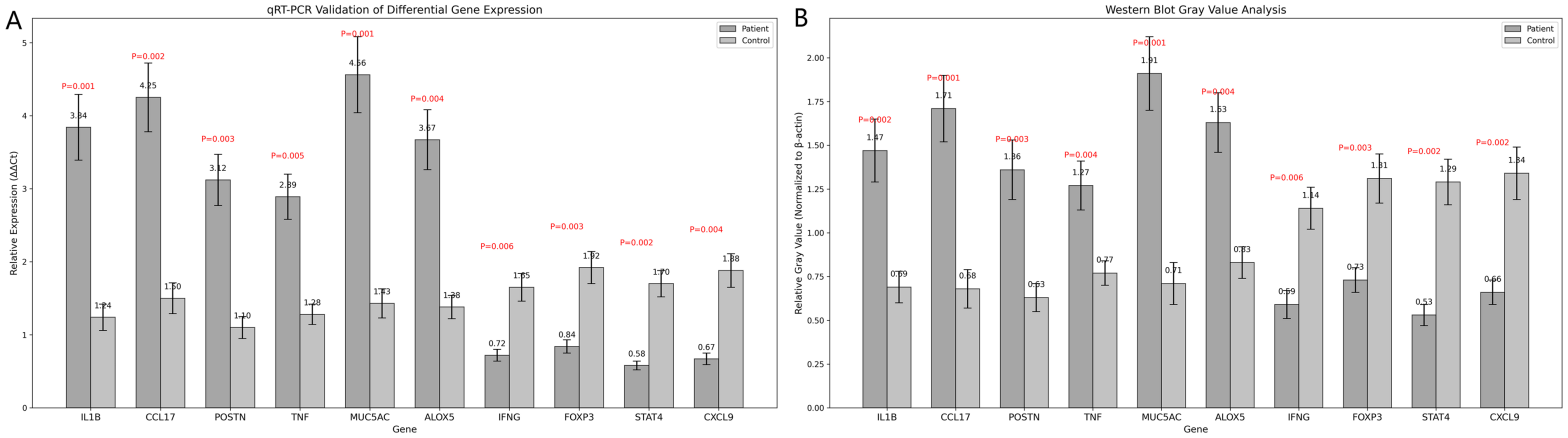
**(A)** Bar chart of qRT-PCR validation results. **(B)** Bar chart of grayscale value analysis from Western blot results. Statistical analysis was conducted using t-tests, with significance evaluated by *P*-values.

qRT-PCR results demonstrated significant differences in the expression levels of 10 candidate genes between the patient and control groups. Genes such as *IL1B* and *CCL17* exhibited significantly upregulated expression in the patient group, while others, like *IFNG* and *FOXP3*, were markedly downregulated (*p* < 0.05), suggesting their potential roles in asthma pathogenesis (Figure 5A).

IHC analysis revealed significantly enhanced staining for several genes in the patient group, including *IL1B* and *MUC5AC*, with scores significantly higher than those of the control group (*p* < 0.05). Conversely, genes such as *FOXP3* and *STAT4* exhibited stronger staining intensity in the control group, indicating distinct roles of these genes in the pathophysiological mechanisms of asthma (Table 4).

**Table 4:** Immunohistochemistry (IHC) scoring results.

Gene name	Mean score (patients)	Mean score (controls)	*P*-value	Effect size (Cohen’s d)
*IL1B*	3.78	1.42	0.002	1.34
*CCL17*	4.12	1.58	0.001	1.45
*POSTN*	3.45	1.33	0.004	1.28
*TNF*	3.22	1.56	0.005	1.15
*MUC5AC*	4.33	1.47	0.001	1.52
*ALOX5*	3.68	1.65	0.003	1.22
*IFNG*	1.28	2.98	0.006	−1.05
*FOXP3*	1.42	3.22	0.004	−1.12
*STAT4*	1.12	3.11	0.003	−1.25
*CXCL9*	1.35	3.45	0.002	−1.38

Independent samples t-tests were used to evaluate significant differences between the patient and control groups, and Cohen’s d was calculated to assess the effect size. Significance was determined using *P*-values.

Western blotting results showed that the expression of multiple target proteins, including *IL1B* and *MUC5AC*, was significantly higher in the patient group compared to the control group (*p* < 0.05). In contrast, *IFNG* and *FOXP3* were significantly upregulated in the control group, suggesting potential differential roles for these proteins in asthma development (Figure 5B).

## 4 Discussion

In this study, whole-transcriptome sequencing analysis revealed significant differential expression of numerous genes between the asthma patient and healthy control groups. Notably, several genes associated with inflammation and allergic responses were either upregulated or downregulated, suggesting their critical roles in the pathogenesis of allergic asthma. The marked upregulation of *IL1B* and *CCL17* indicates that the overexpression of these genes may directly influence immune responses in asthma patients. The upregulation of *IL1B* has been closely linked to an increased release of inflammatory mediators in the airways, activating downstream pro-inflammatory cytokines and exacerbating localized airway inflammation (14, 15). Similarly, the elevation of *CCL17* amplifies the inflammatory response by recruiting Th2 cells, contributing to a persistent state of chronic inflammation in asthma patients. These inflammatory processes are critical to the pathological progression of asthma and may represent a primary cause of airway hyperresponsiveness (16). Beyond airway inflammation, the upregulation of *MUC5AC* highlights its specific role in asthma. *MUC5AC* overexpression leads to increased mucus secretion as a key airway mucin protein. The accumulation of high-viscosity mucus in the airways can obstruct gas exchange, induce airway blockage, and elevate airway resistance, contributing to the hyperreactivity observed in asthma patients (17). These mechanisms provide essential insights into the typical clinical manifestations of asthma, such as shortness of breath and wheezing. Additionally, the downregulation of *IFNG* and *FOXP3* in asthma patients indicates suppression of regulatory immune responses. *FOXP3*, a crucial regulator of regulatory T cell (Treg) development and function, is essential for controlling excessive immune reactions. Its downregulation may reduce regulatory immune cells, impairing the body’s ability to modulate overactive immune responses and rendering inflammation more uncontrollable (18, 19). Similarly, the reduced expression of *IFNG* suggests weakened anti-inflammatory effects of Th1 cells, further skewing the immune response toward a Th2-dominated profile. This imbalance in immune regulation may impair asthma patients’ ability to effectively control their inflammatory processes, exacerbating the symptoms of allergic asthma. The expression patterns of these differentially expressed genes not only elucidate the potential molecular mechanisms underlying asthma pathogenesis but also provide a foundation for identifying targeted therapeutic strategies. In line with our findings, Alhamdan et al. (20) conducted a transcriptomic study on CD4^+^ T cells from asthmatic patients, identifying thousands of differentially expressed genes. They reported significant alterations in interferon (IFN)-related signaling pathways, particularly in obese asthmatics, while gap junction and G protein-coupled receptor (GPCR) ligand-binding pathways were broadly enriched across asthma groups. These findings align with our results, including the downregulation of IFNG in asthma patients, suggesting a shared theme of immune dysregulation. Both studies highlight the critical role of IFN signaling and other inflammatory pathways in asthma pathogenesis. Further exploration of these pathways could advance asthma molecular subtyping and contribute to developing personalized therapeutic strategies.

Functional enrichment analysis of differentially expressed genes demonstrated their involvement in several critical biological processes and signaling pathways in asthma pathogenesis, particularly highlighting their significant role in immune regulation. GO analysis revealed that the differentially expressed genes were enriched in biological processes related to immune response, T cell activation, and inflammatory response, emphasizing the central role of immune responses in asthma development. T cell activation encompassed the recruitment and activation of Th2 cells and the suppression of regulatory T cell functions, consistent with the observed downregulation of FOXP3. This supports the notion of an imbalance in immune regulatory mechanisms in asthma patients (21). KEGG pathway enrichment analysis further identified significant enrichment of the differentially expressed genes in several signaling pathways, particularly the MAPK and PI3K-Akt pathways. These pathways play pivotal roles in airway remodeling, immune cell migration, and the release of inflammatory mediators. Activation of the MAPK signaling pathway enhances the expression of inflammatory factors, contributing to chronic airway inflammation and exacerbating airway hyperresponsiveness in patients (22, 23). Meanwhile, the activation of the PI3K-Akt pathway is closely linked to airway epithelial cell proliferation and structural remodeling, providing a mechanistic explanation for the airway narrowing and hyperreactivity observed in asthma patients. The findings highlight the enrichment of differentially expressed genes in multiple immune and inflammation-related pathways, further reinforcing their central role in asthma pathogenesis. These results provide a clear direction for future research into targeted therapies. Notably, our findings align with recent studies (24–26) highlighting the critical role of epigenetic regulation in asthma pathogenesis. As emphasized by these studies, epigenetic modifications such as DNA methylation, histone acetylation, and non-coding RNAs (e.g., miRNAs and lncRNAs) play a pivotal role in regulating immune and inflammatory responses in asthma. Notably, these mechanisms extend beyond transcriptional regulation, with aberrant epigenetic modifications in leukocytes and airway epithelial cells leading to pronounced alterations in gene expression. Our study observed significant alterations in immune-related pathways, suggesting that epigenetic regulation of immune cells, including T cells, could contribute to asthma development. These findings support the growing evidence that environmental factors influence asthma through epigenetic mechanisms, underscoring the need for further research into these processes. Integrating epigenetic assessments with transcriptomic analyses may provide a more comprehensive understanding of immune imbalance in asthma, ultimately paving the way for improved diagnostics and personalized therapeutic strategies. By exploring the regulatory mechanisms of these key pathways, it is anticipated that more effective and specific treatment strategies for asthma can be developed.

In this study, the differential genes were validated at multiple levels using qRT-PCR, immunohistochemistry (IHC), and Western Blot to ensure the accuracy and reliability of the transcriptomic data. qRT-PCR validation revealed that genes such as *IL1B*, *CCL17*, and *MUC5AC* exhibited significantly higher RNA expression levels in the patient group compared to the control group, while *FOXP3* and *IFNG* were more highly expressed in the control group. These findings were highly consistent with the transcriptome sequencing results, underscoring the transcriptomic data’s reliability and providing a solid foundation for further understanding the roles of these genes in asthma. The results of IHC and Western blot further expanded our understanding of these genes. Western blot analysis showed that the protein expression levels of the target genes were consistent with their transcriptional levels, indicating significant differences in protein expression as well (27, 28). IHC analysis provided detailed information on the spatial distribution of these genes in tissues, revealing their localization in airway epithelial cells and immune cells. This localization is crucial for understanding the specific roles these genes play in the pathophysiology of asthma. Together, the results of these multi-level validation experiments support the critical roles of the identified differential genes in asthma. The consistency of these findings across various biological levels strengthens the scientific foundation of this study and enhances our understanding of the molecular mechanisms underlying asthma pathogenesis.

## 5 Limitations and future directions

This study revealed the roles of several key genes in asthma through whole-transcriptome sequencing and experimental validation. However, the relatively small sample size may limit the generalizability of the findings, particularly in terms of representativeness and statistical robustness. Distinct gene expression profiles may exist in asthma patients with varying disease severities, but this study did not perform detailed subgroup analyses, which restricts a deeper understanding of the molecular mechanisms involved in disease progression. Future research should involve larger, stratified cohorts of asthma patients with different types and stages of the disease to enhance our understanding of the roles of these differential genes. While this study primarily relied on peripheral blood and airway samples, which provided valuable insights into the molecular basis of asthma, the disease involves multiple cell types and a complex microenvironment. Single-cell RNA sequencing could be instrumental in uncovering cellular heterogeneity and delineating the specific roles of individual cell populations in the pathology of asthma. Additionally, functional experiments such as gene knockout and overexpression studies will be critical for further elucidating the biological effects of these key genes in asthma pathophysiology. Such approaches could provide direct evidence for their roles and pave the way for the development of targeted interventions.

## 6 Conclusion

In conclusion, this study underscores the critical role of differential gene expression in the pathogenesis of allergic asthma. Whole-transcriptome sequencing identified key inflammatory genes, such as *IL1B*, *CCL17*, and *MUC5AC*, that were upregulated in asthma patients, while immune regulatory factors like *FOXP3* and *IFNG* were higher in healthy controls. Functional enrichment analysis linked these genes to immune response, T-cell activation, and MAPK signaling pathways. Experimental validation confirmed these findings, highlighting their involvement in immune dysregulation and inflammation, offering potential therapeutic targets. Further research is needed to assess their clinical relevance.

## Data Availability

The data presented in this study are available upon request from the corresponding author (E-mail: xuyanxia@yau.edu.cn). Due to institutional policies, data are not publicly deposited in a repository.
